# Regulatory networks may evolve to favor adaptive foresight

**DOI:** 10.1371/journal.pbio.3002922

**Published:** 2024-12-06

**Authors:** Alejandro Couce

**Affiliations:** Centro de Biotecnología y Genómica de Plantas (CBGP), Universidad Politécnica de Madrid (UPM), Madrid, Spain

## Abstract

Pleiotropy (a single mutation altering many traits) has long been seen as hindering adaptation. A new study in PLOS Biology offers a striking counterexample, suggesting that regulatory networks may evolve to ensure mutations are simultaneously beneficial in correlated environments.

Cells live busy, complicated lives: A vast array of molecules must be managed in an orderly way, while ever-changing conditions need to be closely monitored to prioritize resource allocation across different systems. It is unsurprising, thus, that many genes are pleiotropic; that is, their products influence multiple traits. Naturally, genes involved in sensing and regulatory functions are particularly pleiotropic. In *Escherichia coli*, for example, transcription factor genes account for about 10% of the genome, each one regulating a median of 7 genes (interquartile range: 3 to 15), with nearly 18% regulating more than 20 genes [1]. An inescapable feature of life, pleiotropy has broad implications across biology, including topics such as speciation, development, aging, and human disease [2]. An influential idea is that pleiotropy impedes adaptation. It makes intuitive sense: If most phenotypic changes are typically harmful, then the likelihood of randomly altering multiple phenotypes simultaneously in a beneficial way must be exceedingly low (Fig 1A). This view has led to the long-held expectation that adaptation should primarily occur via small-effect, nonpleiotropic mutations. Moreover, since pleiotropy should scale with the number of traits an organism expresses, adaptation should be much slower in more complex organisms—the so-called “cost of complexity” [3].

**Fig 1:**
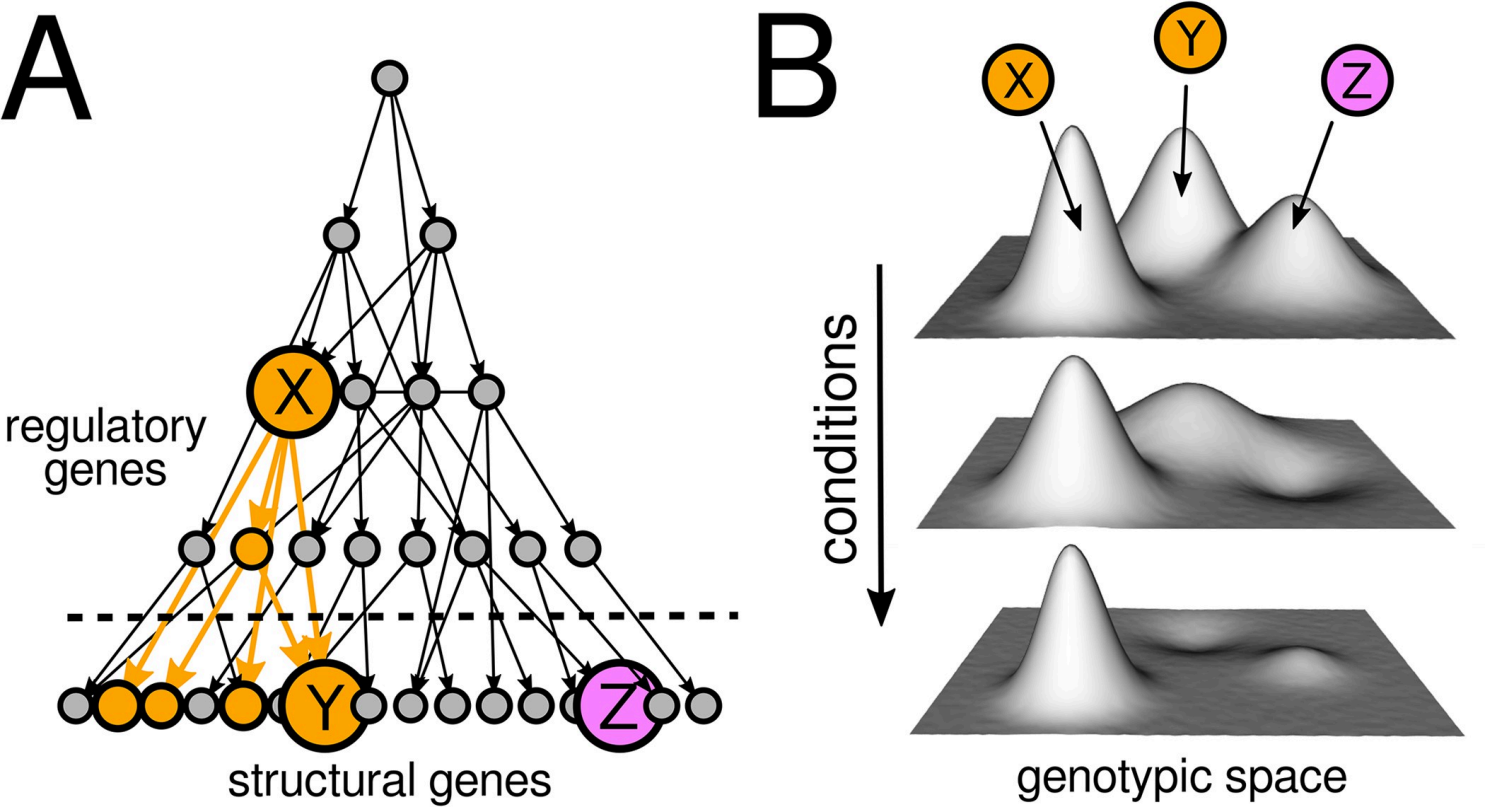
Regulatory genes may be likely to show adaptive pleiotropy. **(A)** Pleiotropic mutations have been typically associated with large deleterious side effects. Mutations in structural genes (Y and Z) should therefore be more likely to drive adaptation than mutations in regulatory ones (X). However, early adaptation in many evolution experiments with microbes is typically driven by large-effect, regulatory mutations. One explanation is that initial adaptation sometimes just requires altering the expression of a specific gene (Y), which can be more easily done through mutations in regulatory genes (X) than by other means. Another explanation is that, under conditions of global stress, mutations in regulatory genes may be the only feasible way to restore basic functionality across the many genes affected simultaneously (yellow, unlabeled circles). **(B)** A new article in *PLOS Biology* suggests a third explanation: regulatory networks may have evolved to generate mutations exhibiting “adaptive pleiotropy”—mutations that influence multiple traits at once in a way that is coherent under correlated selective pressures. In contrast to mutations in structural genes (Y and Z), mutations in an adaptively pleiotropic gene (X) will tend to have beneficial effects across conditions often encountered sequentially in the organism’s natural environment—giving the impression of adaptive foresight (note that in these landscapes, greater height corresponds to higher fitness). While intriguing, further experimental and theoretical work is needed to establish the generality of this phenomenon.

Contrary to these expectations, evolution experiments with microbes recurrently show that the early stages of adaptation often proceed via large-effect, highly pleiotropic mutations [4,5]. Granted, recent theoretical work has challenged the “cost of complexity” hypothesis, pointing out that it relies on the rather extreme assumption that pleiotropy is entirely random and widespread: If a more relaxed view is considered—where pleiotropy is mostly confined to functionally related traits—then pleiotropic mutations may be less costly than previously thought, and, therefore, they could occasionally mediate adaptation [6]. However, the extent to which highly pleiotropic mutations seem to drive adaptive evolution in microbes is striking: nearly all studies where sequencing was applied found multiple mutations in global transcriptional regulators, typically associated with genome-wide changes in expression patterns [4].

Two broad explanations are possible for the prominence of mutations in highly pleiotropic, transcriptional regulators. First, adaptation may sometimes simply require adjusting the activity levels of some enzymes. Disrupting repressors or activators offers a more readily target for this adjustment than fine-tuning promoters or active sites. In *Pseudomonas fluorescens*, for instance, overexpression of a particular operon is the key to enabling sufficient polymer production for colonizing the air-broth interface of a static microcosms. This overexpression is achieved via mutations in regulatory genes, some of which alter the expression of dozens of genes unrelated to the polymer production [7]. Of note, these off-target changes seem deleterious, as suggested by restoration of the expression levels of these genes after further propagation. This 2-step pattern of large-scale transcriptomic rewiring followed by compensatory evolution seems to be a common theme in many microbial evolution experiments [4].

A second explanation for the prevalence of highly pleiotropic mutations pertains to situations of global stress. When many unrelated cellular systems are compromised, single mutations that improve each system individually may not provide sufficient advantage. Under such conditions, regulatory mutations alleviating several crucial bottlenecks simultaneously may be the only route to survival. One of the best examples is mutations in the RNA polymerase, a central hub for coordinating genome-wide gene expression. High-benefit mutations in this enzyme, with global effects in the transcriptome, are commonly observed under intense stress conditions, such as high temperature [8] or starvation [9].

A third, tantalizing possibility is suggested by a new article in *PLOS Biology* [10]: Regulatory networks may have evolved to generate mutations exhibiting “adaptive pleiotropy”; that is, mutations coherently affecting multiple traits at once, particularly traits important for handling correlated selective pressures (Fig 1B). Kinsler and colleagues conducted high-resolution analyses of hundreds of adaptive mutations emerging in yeast under glucose-limited, batch culture conditions. Under these conditions, yeast experience 2 growth phases: first, fermenting glucose into ethanol, and then utilizing that ethanol for respiration. In a prior work [11], they found that approximately 85% of first-step mutants improved both fermentation and respiration, despite these being transcriptomically and physiologically distinct. Interestingly, most of the first-step mutants carried single mutations in the Ras/PKA glucose-sensing pathway. Here, they evaluated whether this adaptive pleiotropy is just a consequence of first-step mutations having much room for adaptation, or whether it is also common among second-step mutants. To this end, they ran a further adaptive step using 5 different backgrounds, each with a unique first-step mutation. Ras/PKA backgrounds typically acquired second-step mutations outside this module, improving respiration alone. Only a background with a first-step mutation in the TOR/Sch9 nitrogen-signaling pathway showed improvements in both fermentation and respiration. Notably, the second-step mutations detected in this background were Ras/PKA mutations, suggesting that adaptive pleiotropy is a unique and generic property of the Ras/PKA glucose-sensing pathway.

How common adaptive pleiotropy is to be expected in general? Yeast growing aerobically on glucose are a special case due to their 2-phase growth strategy, known as the “Crabtree effect.” This phenomenon, evolved after the first fruiting plants appeared, represents a cunning strategy to exploit the sugar-rich niches provided by fruits: Fermentation may be wasteful but it is a rapid way to hog sugars, with the convenient side effect of producing a byproduct (ethanol) that kills your competitors [12]. The physiology and regulation of this strategy have been refined over millions of years of evolution, providing ample opportunity for a major glucose-sensing pathway to develop adaptive pleiotropy. In this context, it would be intriguing to assess whether Crabtree-negative yeast exhibit adaptive pleiotropy when propagated aerobically on glucose.

What about the many examples of regulatory-driven, laboratory adaptation? The prevalence of adaptive pleiotropy is unclear, largely because it is often difficult to determine which traits are relevant to measure. Future work should reexamine some of these examples by recreating mutations in global regulators and characterizing their effects on putatively beneficial independent traits (for instance, entry and exit from lag and stationary phases, maximum growth rate). Additionally, experiments specifically designed to test how readily adaptive pleiotropy can evolve will be most welcome (for instance, evolving under alternating selective pressures that elicit independent transcriptional responses). On the theoretical side, work is needed to understand the conditions under which adaptive pleiotropy can be selected for. As with other instances of second-order selection for evolvability—what this scenario ultimately represents—outcomes are expected to be highly sensitive to multiple parameters. For instance, how sensitive the evolution of adaptive pleiotropy is to fluctuations in the correlation between selective pressures? Are the rates of transcriptional network rewiring enough to sustain this evolution? Moreover, we need a proper null expectation for how readily the natural rewiring of regulatory networks can produce adaptive pleiotropy just by chance. Determining whether adaptive pleiotropy is common and easily selectable will aid in anticipating the evolution of unwanted pathogens and improving biotechnologically-relevant model systems.

## References

[1] Salgado H, Gama-Castro S, Lara P, Mejia-Almonte C, Alarcón-Carranza G, López-Almazo AG, et al. RegulonDB v12.0: a comprehensive resource of transcriptional regulation in *E*. *coli K-12*. Nucleic Acids Res. 2024;52(D1). doi: 10.1093/nar/gkad1072 37971353 PMC10767902

[2] Wagner GP, Zhang J. The pleiotropic structure of the genotype-phenotype map: the evolvability of complex organisms. Nat Rev Genet. 2011;12(3):204–213. doi: 10.1038/nrg2949 21331091

[3] Orr HA. Adaptation and the cost of complexity. Evolution. 2000;54(1):13–20. doi: 10.1111/j.0014-3820.2000.tb00002.x 10937178

[4] Hindré T, Knibbe C, Beslon G, Schneider D. New insights into bacterial adaptation through in vivo and in silico experimental evolution. Nat Rev Microbiol. 2012;10(5):352–365. doi: 10.1038/nrmicro2750 22450379

[5] Ruelens P, Wynands T, de Visser JAGM. Interaction between mutation type and gene pleiotropy drives parallel evolution in the laboratory. Philos Trans R Soc Lond B Biol Sci. 2023;378(1877):20220051. doi: 10.1098/rstb.2022.0051 37004729 PMC10067263

[6] Wang Z, Liao BY, Zhang J. Genomic patterns of pleiotropy and the evolution of complexity. Proc Natl Acad Sci U S A. 2010;107(42):18034–18039. doi: 10.1073/pnas.1004666107 20876104 PMC2964231

[7] Knight CG, Zitzmann N, Prabhakar S, Antrobus R, Dwek R, Hebestreit H, Rainey PB. Unraveling adaptive evolution: how a single point mutation affects the protein coregulation network. Nat Genet. 2006;38(9):1015–1022. doi: 10.1038/ng1867 16921374

[8] Tenaillon O, Rodríguez-Verdugo A, Gaut RL, McDonald P, Bennett AF, Long AD, Gaut BS. The molecular diversity of adaptive convergence. Science. 2012;335(6067):457–461. doi: 10.1126/science.1212986 22282810

[9] Conrad TM, Frazier M, Joyce AR, Cho BK, Knight EM, Lewis NE, et al. RNA polymerase mutants found through adaptive evolution reprogram *Escherichia coli* for optimal growth in minimal media. Proc Natl Acad Sci U S A. 2010;107(47):20500–20505. doi: 10.1073/pnas.0911253107 21057108 PMC2996682

[10] Kinsler G, Li Y, Sherlock G, Petrov DA. A high-resolution two-step evolution experiment in yeast reveals a shift from pleiotropic to modular adaptation. PLoS Biol. 2024;22(12):e3002848. doi: 10.1371/journal.pbio.3002848 39636818 PMC11620474

[11] Li Y, Venkataram S, Agarwala A, Dunn B, Petrov DA, Sherlock G, Fisher DS. Hidden complexity of yeast adaptation under simple evolutionary conditions. Curr Biol. 2018;28(4):515–525.e6. doi: 10.1016/j.cub.2018.01.009 29429618 PMC5823527

[12] Pfeiffer T, Morley A. An evolutionary perspective on the Crabtree effect. Front Mol Biosci. 2014;1:17. doi: 10.3389/fmolb.2014.00017 25988158 PMC4429655

